# Polymorphous Low-Grade Adenocarcinoma of the Tongue Base Treated by Transoral Robotic Surgery

**DOI:** 10.1155/2015/981436

**Published:** 2015-04-14

**Authors:** Jeong Hong Kim, Chang Lim Hyun, Gil Chai Lim

**Affiliations:** ^1^Department of Otolaryngology Head-Neck Surgery, Jeju National University School of Medicine, Jeju City 690-767, Republic of Korea; ^2^Department of Pathology, Jeju National University School of Medicine, Jeju City 690-767, Republic of Korea

## Abstract

Polymorphous low-grade adenocarcinoma is a rare malignancy arising from the minor salivary glands in the aerodigestive system, most frequently the hard palate. The treatment of choice is wide surgical resection, and the efficacy of radiotherapy has not been confirmed. A 54-year-old male presenting with a mass at the base of the tongue performed transoral laser microsurgery. The pathologic diagnosis was polymorphous low-grade adenocarcinoma. Complete surgical excision was performed via transoral robotic surgery without a flap reconstruction of the surgical defect. Without complications of bleeding or injury to the hypoglossal nerve, proper surgical margins were obtained, and no recurrence was found after 6 months after surgery. The patient did not complain of dysphagia or aspiration. We conclude that, in surgery for tongue base tumors with unknown malignant potential, transoral robotic surgery can be considered for achieving a definite resection avoiding a mandibulotomy without complications of dysphagia or aspiration after confirmation of malignancy with a frozen biopsy.

## 1. Introduction

In 1984, polymorphous low-grade adenocarcinoma (PLGA) was first defined by Evans and Batsakis as a malignant tumor arising in the minor salivary glands previously reported as lobular carcinoma or terminal duct carcinoma [1]. PLGA shows structural diversity upon pathological examination, including solid, cribriform, tubular, trabecular, fascicular, and papillary patterns, but uniform cytology of myoepithelial or luminal duct cells [2]. PLGA is the second most common malignancy in the minor salivary glands, with the hard palate being the most frequently involved site of the head and neck [3]. A female predominance has been reported, with a female : male ratio of 2–4.6 : 1 [4, 5], and the highest incidence is found in the sixth and seventh decades of life [5]. PLGA has been previously reported at the paranasal sinus, nasopharynx, base of the tongue, and larynx [6–8]. Since its introduction by O'Malley and colleagues in 2006 [9], transoral robotic surgery (TORS) has been recognized as an efficient method for resection of the tongue base tumors, largely due to the adequate visual field and three dimensional images offered while avoiding the risks associated with mandibulotomy. We here report a case of PLGA at the base of the tongue diagnosed by surgical excision with transoral laser microsurgery (TLMS) and widely excised via TORS.

## 2. Case Presentation

**Figure 1:**
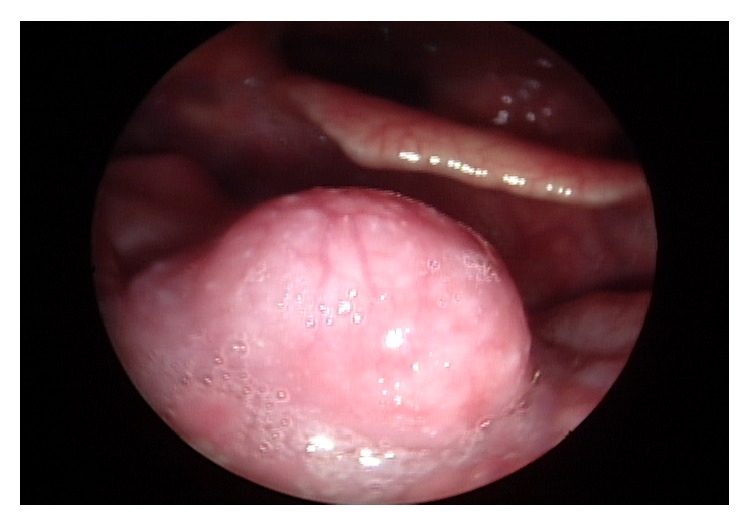
Laryngoscopy of a 54-year-old man with an incidentally detected mass at the tongue base. The tumor was 1 cm in size, and the overlying mucosa was not invaded.

**Figure 2:**
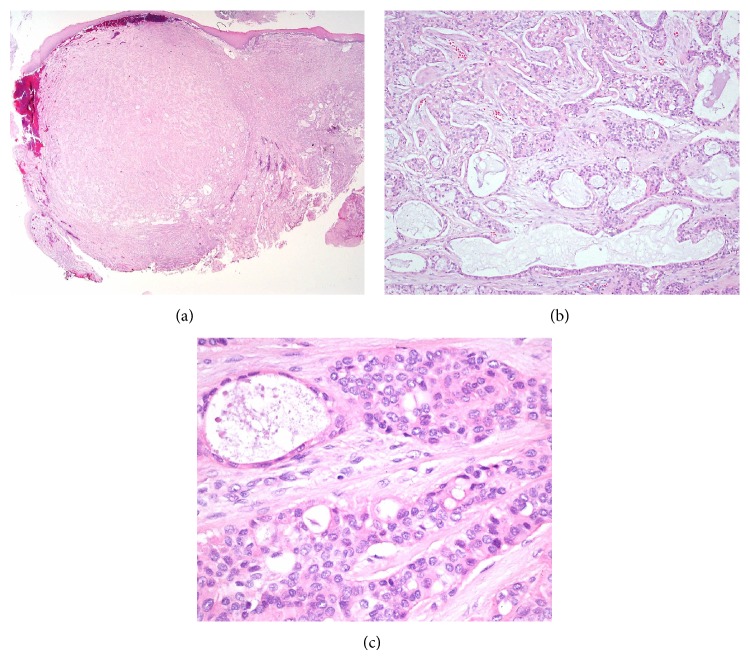
Histologic findings of the tumor. Low-power magnification view, hematoxylin and eosin staining, ×10, showed a well-circumscribed, but not encapsulated, mass with a variety of different growth patterns (a). Variable architectures were seen within tumor tissue, including tubular, trabecular, and cribriform growth patterns, hematoxylin and eosin staining ×100. The tumor cells were small-to-medium sized and uniform in shape (b). The nuclei of the tumor cells were round to oval, with vesicular pale nuclear chromatin and small nucleoli. The mitotic figures were inconspicuous, hematoxylin and eosin staining ×400 (c).

**Figure 3:**
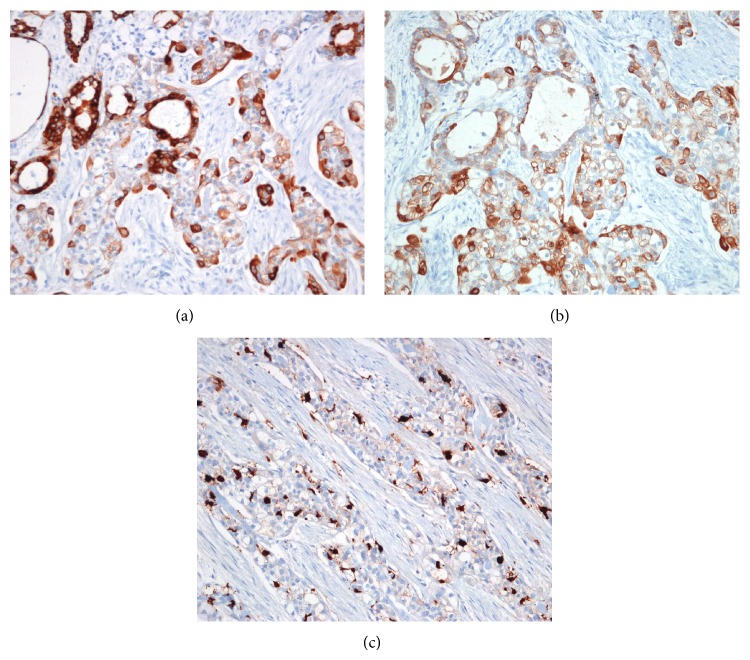
Immunohistochemistry of the tumor. Immunohistochemical analysis revealed that the tumor cells were strongly positive for cytokeratin 7 (a) and epithelial membrane antigen (EMA) (b). A focal staining pattern was shown for S-100 (c).

A 54-year-old male presenting with a mass at the base of the tongue, incidentally found during a routine gastroscopy, visited the outpatient clinic of the otolaryngologic department. He had no complaints of symptoms such as odynophagia or dysphagia. Upon rigid laryngoscopic exam, a tumor, 1 cm in size with intact overlying mucosa, was found at the midline of the tongue base (Figure 1). There was no palpable cervical lymphadenopathy. For pathologic diagnosis and treatment, the patient underwent TLMS. During surgical excision, the mass was found to be hard and well-circumscribed, but the overlying mucosa was not clearly distinguished from the surrounding normal mucosa. The pathologic diagnosis was PLGA with extensive clear cell change, and the resection margins were positive with tumor cells. The lesion was well-circumscribed, but not encapsulated with different growth patterns, such as tubular, trabecular, and cribriform patterns, and the tumor cells were uniform in shape (Figure 2). Upon immunohistochemistry, positive expression of cytokeratin, epithelial membrane antigen (EMA), and S-100 protein were demonstrated (Figure 3). Postoperative enhanced computed tomography (CT) scan and positron emission tomography (PET) revealed a 1 cm sized, elliptical shaped cervical lymphadenopathy in right level II that had insignificant standardized uptake value (SUV). Fine needle aspiration cytology via ultrasonography did not show metastatic spread.

**Figure 4:**
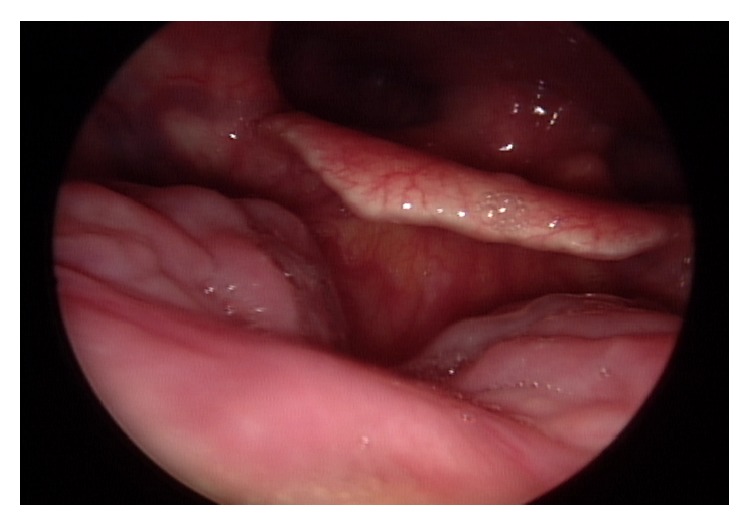
Laryngoscopy at the 6-month follow-up. No evidence of tumor recurrence was found.

For definite surgical excision, transoral robotic surgery (TORS) with the da Vinci robotic system (Intuitive Surgical, Sunnyvale, CA, USA) was performed one month after the initial surgery. A McIvor mouth retractor was placed for access of the 30° telescope and two robotic arms. ProGrasp forceps and a monopolar spatula were applied at the two robotic arms. About 1 cm sized free mucosal margins were obtained from the previous excision site, and the muscle of the tongue base was resected with 1 cm sized depth. During TORS, there were no injuries to the lingual artery and hypoglossal nerve. Frozen biopsies of the resection margins were free of tumor cells, and reconstructive flap surgery was not necessary. The patient did not complain of dysphagia or pulmonary aspiration in the immediate postoperative periods. Upon laryngoscopic examination 6 months after TORS, there was no evidence of tumor recurrence (Figure 4), and the patient had not suffered from dysphagia or aspiration since the surgery.

## 3. Discussion

Common malignancies arising from the minor salivary glands include, in order of appearance, adenoid cystic carcinoma, mucoepidermoid carcinoma, and adenocarcinoma with adenocarcinomas representing 20–25% of all malignant salivary gland tumors in the tongue [4]. The incidence of PLGA has not been reported specifically, but the oral cavity especially the hard palate is known to be frequently involved. PLGA is mainly found at the base of the tongue, but in case of anterior tongue involvement has been reported [10].

Histologic characteristics of PLGA include morphological diversity and cytologic consistency and invasive growth patterns to surrounding structures [2]. In general, PLGAs have well-circumscribed contours, but no capsular formation, and the overlying mucosa is not invaded by tumor [11]. Distinguishing PLGA from pleomorphic adenoma or adenoid cystic carcinoma is important for accurate pathologic diagnosis. Grossly, pleomorphic adenoma has a well-circumscribed margin and capsular formation and, microscopically, chondromyxoid matrix and plasmacytoid cells are noted. The differential diagnosis of adenoid cystic carcinoma is more challenging, but can be confirmed by identifying cribriform and tubular structures with double-layered ducts composed of angular hyperchromatic cells [12]. There are currently no demonstrated, unique immunohistochemical markers for PLGA [13].

PLGA is very rarely associated with regional and distant metastasis. Several studies have reported the incidence of regional and distant metastasis as between 5 and 15% [1, 14] and 0.6 and 7.5%, respectively [1, 11]. The nature of the disease is generally indolent, and the prognosis is relatively good. Wide surgical resection is recommended as a primary treatment, and the role of radiotherapy is controversial. In patients who have close or positive surgical margins, a postoperative radiation therapy could be considered based on weak evidence [12]. Therefore, an elective neck dissection is not recommended in early T stage tumors [12, 14]. In our patient, there was a cervical lymphadenopathy that was detected by CT and PET scans; however, characteristics of this lymphadenopathy could be thought not to be benign in radiological evaluations. The fine needle aspiration cytology also showed no metastasis; as a result, we decided not to perform an elective neck dissection.

In surgery of the base of the tongue, the transoral approach is limited by the poor visualization of the surgical field and the acquirement of adequate surgical margins. Moreover, bleeding control is difficult in this approach, and mandibulotomy is thus generally performed in tongue base surgery. Because TLMS for tongue base tumors is interfered owing to the straight pattern of laser, TORS may be more effective in this area due to the angled telescope and bendable robotic arms in achieving “en bloc” resection for tumors, even if more than 1 cm size. In TORS, obtaining safe surgical margins, controlling bleeding of the lingual artery, and preserving the hypoglossal nerve can be more easily achieved than in TLMS [15]. In our case, TLMS was performed initially, because the size of the tumor was small and thought to be a benign disease. Initial TLMS for the tumor was not a difficult procedure; however, TORS was thought to be necessary to achieve more definite tumor removal. By using this strategy, we could avoid mandibulotomy and were able to remove sufficient tongue base tissue without causing massive bleeding of the lingual artery or injury to the hypoglossal nerve.

## 4. Conclusion

We conclude that in surgery for tongue base tumors with low-grade malignant potential, transoral robotic surgery can be considered for achieving a definite resection avoiding mandibulotomy without complications of dysphagia or aspiration. Additionally, transoral robotic surgery could be considered for a treatment of choice in tongue base tumors with unknown malignant potential after confirmation of malignancy with a frozen biopsy.
